# Heterelogous Expression of Plant Genes

**DOI:** 10.1155/2009/296482

**Published:** 2009-08-06

**Authors:** Filiz Yesilirmak, Zehra Sayers

**Affiliations:** Faculty of Engineering and Natural Sciences, Sabanci University, Orhanli, Tuzla, 34956 Istanbul, Turkey

## Abstract

Heterologous expression allows the production of plant proteins in an organism which is simpler than the natural source. This technology is widely used for large-scale purification of plant proteins from microorganisms for biochemical and biophysical analyses. Additionally expression in well-defined model organisms provides insights into the functions of proteins in complex pathways. The present review gives an overview of recombinant plant protein production methods using bacteria, yeast, insect cells, and *Xenopus laevis* oocytes and discusses the advantages of each system for functional studies and protein characterization.

## 1. Introduction

Heterologous expression involves identification of genes and transfer of the corresponding DNA fragments to hosts other than the original source for synthesis of the encoded proteins. Protein isolation, especially from plant sources, can be costly, cumbersome and lengthy, and heterologous expression provides a convenient alternative. This methodology allows large-scale production of plant proteins in microorganisms to study their biochemical and biophysical features. Foreign hosts may also provide a simpler system for studies on functions of proteins and for elucidation of their roles in complex mechanisms such as metabolic reactions and membrane transport. Recombinant plant proteins and peptides produced by heterologous expression are also used in industrial applications. Examples are provided by the synthesis of a medicinal peptide from ginseng as potential drug against diabetes [1] or production of plant lectins [2] in both cases in yeast. 

The present review covers the recent literature on plant gene expression in bacteria, yeast, insect cells and *Xenopus* oocytes and presents the comparative advantages and disadvantages of each system. It also provides a survey of recent examples of application of heterologous expression technology to plant proteins. A comprehensive list of plant proteins expressed heterologously is given in Table 1. Factors influencing the choice of hosts, including the stability and folding characteristics of the protein, requirement for posttranslational modifications, efficiency of the expression system, as well as simplicity and cost are discussed in the following sections.

## 2. Principal Components of Heterologous Expression

**Figure 1:**
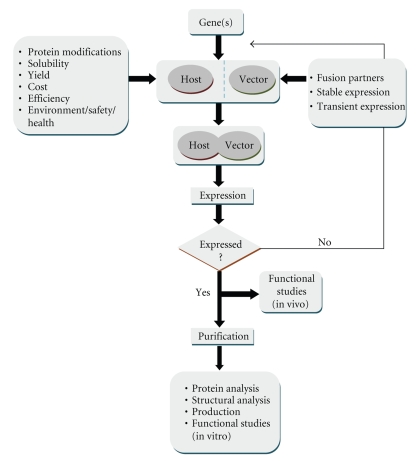
Flow chart for heterologous expression.

Basic principles of heterologous cloning and expression are summarized in Figure 1. Major parameters that affect choices at different stages are also indicated. The choice of the expression system and vector is a critical step in this procedure and, as indicated, advantages and disadvantages of several factors have to be considered. Expression systems are selected depending on whether the purpose of study is production of large quantities of protein or investigation of functional features of the cloned protein. The physicochemical properties of the investigated protein also play a role in this choice. A general review of frequently used expression systems is provided by Yin et al. [3]. 

A comprehensive survey of commercially available expression vectors has recently been published [4]. The most commonly used vectors are fusion systems that link additional amino acid sequences (tags) to the protein through a recognition site for a specific protease. Tags may consist of a short peptide sequence or a full protein which can be cleaved from the protein when desired. Presence of tag sequences facilitates solubility, purification, quantification, identification, localization, and assaying of the expressed protein. Frequently used fusion partners include glutathione-S-transferease (GST), his-tag (poly-histidines), maltose binding protein (MBP), thioredoxin (TrxA), FLAG epitope-tag, c-Myc epitope-tag, disulfide isomerase I (DsbA), polyarginine-tag (Arg-tag), calmodulin-binding peptide, cellulose-binding domain, poly-histidine affinity tag (HAT-tag), N-utilizing substance-A (NusA), S-tag, streptavidin-binding peptide (SBP-tag), strep-tag, fluorescent proteins (e.g., green fluorescent protein (GFP)) and ubiquitin [4]. MBP and NusA are specifically used to increase the solubility. MBP is considered to be much more effective for enhancing solubility than GST and thioredoxin [5]. The major disadvantages of fusion protein systems are the requirement of expensive proteases for cleavage from the recombinant protein and the low yield of cleavage reactions [6]. 

Depending on the host system, vectors for transient or stable expression can be chosen as indicated below.

## 3. Expression Hosts

### 3.1. Prokaryotic Expression Systems

#### 3.1.1. Escherichia Coli

*Escherichia coli* (*E. coli*) is the first and most extensively used prokaryotic expression system for heterologous protein production [7]. It remains generally the first choice due to its simplicity, rapid growth rate, and relatively low cost. Almost all commercially available inducible cloning vectors are compatible with *E. coli* and extensive biochemical and genetic information is available. 

One of the disadvantages of using *E. coli* as an expression host arises from its inability to perform post-translational modifications, which are often required for correct folding and functional activity of the recombinant protein. This applies particularly to some membrane proteins and enzymes [3]. Another disadvantage is that *E. coli* is generally not suitable for proteins which contain many disulfide bonds or require glycosylation, proline cis/trans isomerization, disulfide isomerization, lipidation, sulphation, or phosphorylation [8]. Some eukaryotic proteins that retain their full biological activity in the nonglycosylated form have, however, been produced in *E. coli.* The unglycosylated human growth hormone (hGH) binding protein secreted from *E.coli* retains the same binding affinity and specificity as the wild-type hGH binding protein suggesting that recombinant protein is properly folded and glycosylation is not required for binding [9]. 

Production of proteins that are stabilized by disulfide bonds in *E.coli * often results in proteolytic degradation or misfolding and formation of inclusion bodies [6]. One strategy developed to improve this situation is to target these proteins to the periplasm where the nonreducing environment allows formation of disulfide bonds [10, 11]. In addition, the *E.coli* periplasm contains chaperone-like disulfide-binding proteins (DsbA, DsbB, DsbC, and DsbD), folding catalysts, and peptidyl-prolyl isomerases (SurA, RotA, FklB, and FkpA) that support disulfide bond formation and are important for correct folding of periplasmic proteins [12–14]. Disulfide bond formation is achieved via fusion to DsbA or DsbC [15, 16] and periplasmic secretion results in the functional production of a variety of recombinant proteins [17]. In a recent study, the rescue of unstable lipase B from *Pseudozyma antarctica* (PalB), with periplasmic folding factors was demonstrated [18]. Another strategy involves the use of the *trx*B *gor* double mutant lacking thioredoxin reductase and glutathione reductase genes [19, 20]. This double mutant was used for heterologous expression of barley oxalate oxidase (HvOXO) in *E.coli* [21]. The gene for an osmotin-like cryoprotective protein from *Solanum dulcamara* was expressed in *E.coli* and directed to periplasmic localization using an expression vector containing the *pelB* signal sequence [22]. This resulted in high concentrations of soluble protein with cryoprotective activity, whereas expression in the bacterial cytoplasm only yielded large amounts of insoluble and aggregated protein. 

Some of the plant proteins accumulated in insoluble inclusion bodies in *E.coli* can be solubilized and refolded to restore activity after purification from the host. Examples include Arabidopsis thaumatin-like protein (ATLP3) which was purified from inclusion bodies and the refolded form displayed activity against some pathogenic fungi [23]. To validate the potential antifungal activity of *Solanum nigrum* osmotin-like protein (SnOLP) was overexpressed in *E.coli* and the recombinant protein was refolded using reduced:oxidized gluthatione redox buffer and its in vitro activity was demonstrated [24]. The soybean RHG1-LRR domain protein was solubilized from inclusion bodies using urea and refolded by removing the urea in the presence of arginine and reduced/oxidized glutathione [25].

Many plant enzymes are expressed in insoluble inclusion bodies but it is still possible to obtain high yields of active forms for structural studies [26]. The mature polypeptide of FatB thioesterase from the developing seed tissues of *Madhuca butyracea* was characterized by heterologous expression in *E.coli* [27]. The functionality of the MbFatB in the heterologous system was revealed by the altered growth behavior and cell morphology of the bacteria due to the changes in the fatty acid profile. The maize chloroplast transglutaminase (TGZ) [26] and glutamatecysteine ligase (GCL) [28] were efficiently overexpressed in *E.coli*. Recently, DELLA proteins from both Arabidopsis and *Malus domestica,* which are involved in regulation of plant growth in response to phytohormonal signals, were isolated and expressed in *E. coli* [29].

Examples of functional expression of plant proteins in *E.coli* are provided mostly by studies on membrane proteins. A mutant with very low K^+^ uptake was used as host for studies on the K^+^ transporters AKT2 [30], AtKUP1-2 [31], AtHKT1 [32] from Arabidopsis and EcHKT1 and EcHKT2 from *Eucalyptus camaldulensis* [33]. In another example, *E.coli* C43 strain, which is suitable for expression of membrane proteins was used for functional characterization of chloroplast ATP/ADP transporter from Arabidopsis [34]. The seagrass HAK K^+^ transporters, CnHAK1 and CnHAK2 were also overexpressed in *E.coli* and it was found that CnHAK1, but not CnHAK2, mediated very rapid K^+^ or Rb^+^ influxes [35]. Using a dicarboxylate uptake-deficient *E.coli * mutant, a peptide transporter, AgDCAT1 from alder, was shown to be a dicarboxylate, including malate, succinate, fumarate, and oxaloacetate, transporter [36].

**Figure 2:**
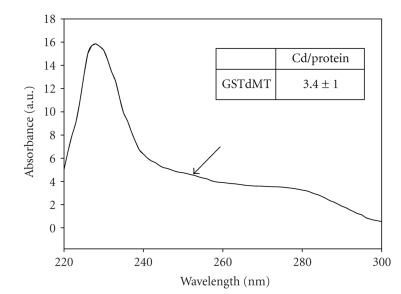
UV-visible absorption spectrum of GSTdMT at 2.7 mg/mL concentration in 20 mM HEPES buffer at pH 8.0. The charge transfer band between 240 and 260 nm due to Cd-S interaction is indicated by the arrow. The Cd/protein ratio is given in the inset.

**Figure 3:**
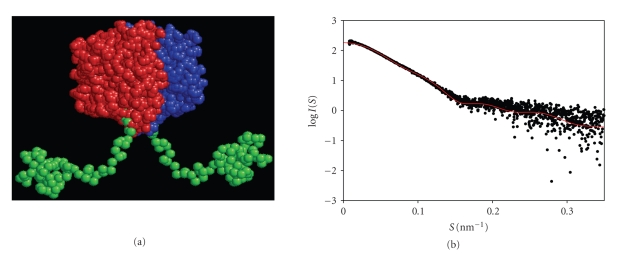
A: a low resolution shape model for GSTdMT. The GST dimer (red and blue) is located at the center from which the dMT molecules extend (green). B: the scattering curve expected from the model (*–*) agrees well with the experimental data (*…*). I(S) is the scattering intensity and S the scattering vector given by S = 4*π*sin*θ*/*λ*, where 2*θ* is the scattering angle and *λ*= 1.5 Å is the wavelength of X-rays. The model and the expected scattering pattern were calculated using the programs in the ATSAS package (EMBL Hamburg Outstation).

*E.coli* has also been used for expression of small plant proteins with a fusion partner. Metallothioneins (MTs), which are difficult to purify from natural sources because of their small molecular weight (7 kD), unusual amino acid sequences containing a large number of cysteins and their proteolytic susceptibility belong to this class. Several MTs including a Cd^2+^ binding Type 1 durum wheat metallothionein (dMT) [37], fava bean Type 1 and Type 2 MTs [38], Arabidopsis MT1, MT2 and MT3 proteins [39], Type 3 MT3-A from the oil palm [40], Type 2 MT, QsMT from *Quercus suber* [41] have been produced in *E.coli* mainly for structural analyses. Since the fusion constructs of durum MT with GST (GSTdMT) can be purified in well-defined oligomeric states they are used as model systems for studies on metal-binding and for structural analyses. Figure 2 illustrates that Cd-binding to GSTdMT can be detected by UV-visible spectroscopy. The metal content of GSTdMT was shown to be the same as that expected from dMT alone. An example of the shape models generated from X-ray solution scattering data for GSTdMT is shown in Figure 3, together with the fit to experimental data. The models support a fold for dMT similar to that expected for the free molecule [42, 43]. These results are in agreement with earlier work suggesting independent folding of GST and its fusion components [44] and indicate that recombinant fusion complexes are useful as model systems for structural studies. 

### 3.2. Eukaryotic Expression Systems

Eukaryotic expression systems offer the possibility of posttranslational modifications and are often used for investigations of protein function. Processing reactions such as *O-*and *N*-linked glycosylation, tyrosine, serine, and threonine phosphorylation, addition of fatty acid chains, processing of signal sequences, disulfide bond formation, and correct folding can all be readily performed in eukaryotic hosts. The most commonly used eukaryotic systems are yeast, insect, mammalian, and plant cells. 

#### 3.2.1. Yeast

As a single cell eukaryotic organism, yeast has molecular, genetic, and biochemical characteristics which are similar to those of higher eukaryotes, and is useful for heterelogous protein production. Yeast cells can grow rapidly with high cell densities, and are easy to manipulate and yeast cultures are cost effective. The two most commonly used organisms are *Saccharomyces cerevisiae* (*S. cerevisiae*) and *Pichia pastoris (P. pastoris)* [7].

**Saccharomyces Cerevisiae**

Baker's yeast, * S. cerevisiae*, is widely used as a host organism for heterologous expression of proteins. Its genetics and physiology are well documented and proteins are posttranslationally modified through the mechanisms similar to those found in plants. The limitations of this host system are low yields, cell stress due to the presence of the foreign gene and hyperglycosylation of secreted foreign proteins. Lack of a strong inducible promoter can be circumvented using *P. pastoris* [45].

Earlier work on heterelogous expression for screening of plant cDNA libraries by complementation in *S. cerevisiae* null mutants was reviewed by Frommer and Ninnemann [7]. The *S. cerevisiae * mutants provide a convenient system for functional and kinetic studies of transporters [46]. The electrophysiological properties of membrane transporters, H^+^-amino acid symporter and K^+^ channel, KAT1 [47] and phosphate transporters; AtPT1 and AtPT2 of Arabidopsis were characterized using *S. cerevisiae* [48]. Recently, functional expression of transporters such as an HvHAKI from barley [49], AtKT1 and AtKT2 [50], and AtKUP1 from Arabidopsis [51] also utilized *S. cerevisiae* mutants. Another K^+^ transporter characterized in this system is HKT1 from wheat [52, 53]. Kinetic uptake analyses of tomato sulfate transporters, LeST1-1 and LeST1-2 were carried out using the *S. cerevisiae* sulfate transporter mutant [54]. The five members of the copper transporter family COPT1–5 from Arabidopsis were characterized using a copper transport null mutant [55]. A peptide transporter *AtPTR1* gene from Arabidopsis was isolated and complemented in a peptide transport-deficient mutant [56]. A putative K^+^/H^+^ antiporter, *At*Chx17 was heterologously expressed and characterized in an *S. cerevisiae kha1 * deletion mutant [57]. To test their functional activity, the grapevine hexose transporters VvHT3, VvHT4, and VvHT5 were expressed in the *S. cerevisiae* mutant EBY.VW4000, which is deficient in glucose transport due to concurrent knock-out of 20 endogenous transporter genes [58]. Growth-based complementation assays were used to demonstrate function of the transporters but resulted in inadequate rates of glucose uptake. A more sensitive assay based on direct measurement of radioactively labelled glucose uptake revealed that this mutant expressing VvHT4 and VvHT5 accumulated labelled glucose at higher rates than yeast transformed with the empty vector, demonstrating the functionality of the glucose transporters. Although VvHT3:GFP (green fluorescent protein) fusion protein was targeted to the plasma membrane in plant cells, VvHT3 was found not to be functional in the yeast system [58].Yeast expression studies were, in several instances, complemented by studies in other organisms to verify functional and kinetic properties of recombinant proteins. The plasma membrane-localized H^+^/inositol symporter AtINT2 of Arabidopsis was studied by expression in an inositol uptake/inositol biosynthesis double mutant in *S. cerevisiae* and in *Xenopus* oocytes [59]. In this study, the amount of AtINT2 protein in yeast plasma membrane was sufficient for complementation, but not for functional and kinetic analyses. In oocytes, however, it was possible to show that AtINT2 mediated the symport of H^+^ [59]. Expression and functional characterization of Arabidopsis AtGAT1 in *S. cerevisiae* and *Xenopus* oocytes revealed that AtGAT1 mediates H^+^-dependent, high affinity transport of high affinity *γ*-aminobutyric acid (GABA) and GABA-related compounds. Properties of this protein could be examined in more detail in *Xenopus* oocytes [60]. Heterologous expression of AtTIP2;1 and AtTIP2;3 from Arabidopsis in both ammonium uptake-defective yeast and oocytes indicated that these TIPs transport both ammonium and methyl-ammonium in addition to water and urea [61]. The kinetic characteristics of the sorbitol transporters, PmPLT1, and PmPLT2 from common plantain (*Plantago major*) were investigated by functional expression in *S. cerevisiae* and in *Xenopus oocytess*. In the yeast system, both proteins were characterized as low-affinity and low-specificity polyol symporters. These data were confirmed in the *Xenopus* system, where PmPLT1 was analyzed in detail and characterized as an H^+^ symporter [62].

The major disadvantages of using *S. cerevisiae* mutants in transporter studies are the hyperpolarization of the membrane, mislocalization of membrane proteins and recruitment of non-K^+^-transporters into K^+^-transporters [63].

**Pichia Pastoris**

*P. pastoris*, methylotrophic yeast, is considered a valuable tool for high yield heterologous expression of various proteins. The possibility of obtaining posttranslational modifications, high level expression of foreign proteins in either intracellular or extracellular forms, simplicity of genetic manipulations, and availability of various *P. pastoris* strains and vectors make this expression system highly popular [64]. Molecular manipulations such as gene targeting, high frequency DNA transformation, and cloning for functional complementation are similar to those in *S. cerevisiae* [64]. Tightly regulated promoters, easy integration of heterologous DNA into the host chromosome and the capacity to generate more posttranslational modifications make *P. pastoris* the preferred system compared to *S. cerevisiae*.

The wide use of *P. pastoris* expression system for recombinant plant proteins can be seen from recent reviews [64, 65]. *P. pastoris* is particularly well suited for studying plant enzymes since glycosylation of the foreign proteins is expected to be closer to that in plants [66, 67] and glycosylated proteins have shorter glycosyl chains in * P. pastoris * than in *S. cerevisiae* [68]. This expression system has the potential to produce high levels of recombinant proteins [67], up to 400 mg/L of culture [69]. Several plant enzymes have been produced in *Pichia. * Two examples are cytosolic expression of nitrate reductase from spinach and corn at high levels needed for detailed biochemical studies [69] and expression of a sweet potato invertase in milligram quantities [70]. Enzymatic activity of the membrane-bound *α*1,6-galactosyltransferase was shown through overexpression in *P. pastoris* [71]. The hypothesis that *α*-xylosyltransferase is involved in xyloglucan biosynthesis was tested by overexpressing the corresponding genes and identifying the gene product that displayed activity [72]. *P. pastoris * has been used for production of a number of glycosyltransferases involved in the biosynthesis of *N*- and *O*-linked oligosaccharides [73]. To confirm that *Os*β*fruct3* from rice encoded a vacuolar type *β*-D-fructofuranosidase, the *Os*β*fruct3* cDNA was expressed in this host [74]. A recombinant potato apyrase was expressed and purified in the hyperglycosylated form at 1 mg/L protein concentration [75]. The catalytically active barley oxalate oxidase, HvOXO was produced with a yield of 50 mg/L culture and biochemically characterized [76].

High-level expression of wheat germin/oxalate oxidase was achieved in *P. pastoris *as an *α*-mating factor signal peptide fusion to increase secretion of the protein of interest into the culture medium. Approximately 1 g (4 × 10^4^ U) of TaOXO was produced in 5 L fermentation cultures following 8 days of methanol induction, demonstrating the possibility of large-scale production of oxalate oxidase for biotechnological applications. Glycosylation of the recombinant protein was evidenced by mass spectrometry [77]. Another application using *P. pastoris* is the expression of the *α*-subunit of heterotrimeric G-proteins, GPA1, from Arabidopsis. Several attempts had previously failed to produce this protein in *E. coli*, whereas in the yeast system the protein could be expressed with a his_6_-tag and purified by affinity chromatography with a yield up to 20 mg from 700 mL culture [78].

Several allergens including, Cyn d 1 from Bermuda grass, Bla g 4 from *German cockroach*, Amb a 6 from *Ambrosia artemisiifolia,* and Ole e 1 from *Olea europaea* have also been produced in *P. pastoris * (see list in 64).

This system was also used for the expression of a number of plant lectins such as *Canavalia brasiliensis* lectin (ConBr) [2] and the *Nicotiana tabacum* lectin [79]. In a recent study, the low-affinity cation transporter (LCT1) from wheat was also expressed and functionally characterized using *P. pastoris* [80].

#### 3.2.2. Insect Cells

Baculoviruses have been used for the synthesis of a wide variety of eukaryotic recombinant proteins in insect cells. In this expression system one of the nonessential viral genes is replaced with the target protein through homologous recombination. The resulting recombinant baculovirus is used to infect cultured insect cells and the heterologous genes can be expressed under the control of the extremely strong pPolh, polyhedron promoter in the late phase of infection. 

The most common baculovirus used for expression studies is *Autographa californica* multiple capsid nucleopolyhedrovirus (*Ac*MNPV) and the most frequently used host insects are *Spodoptera frugiperda* and *Trichoplusia ni*. This expression system produces high levels of recombinant proteins which are soluble, post-translationally modified, biologically active, and functional [81]. The virus is not pathogenic to vertebrates or plants. The main drawback of this system over the bacterial and yeast systems lies in the noncontinuous expression of the heterologous gene; every round of protein production needs reinfection [3].

This heterologous expression system is mainly used to investigate enzymatic mechanisms in plants. The most recent examples include the Arabidopsis reductase isoforms, AR1 and AR2 [82], peroxisomal short-chain acyl-CoA oxidase A [83], cyclin-dependent kinase A [84], NADH-cytochrome b5 reductase [85], geranylgeranyltransferase-I [86], acyl-CoA synthetase [87], homogentisate phytyltransferase [88], (+)-abscisic acid 8’-hydroxylase [89], *β*1,2-xylosyltransferase [90], tobacco ethylene-inducing xylanase [91], and barley ADP-glucose pyrophoshorylase [92]. The overall yield of heterelogous proteins obtained with this system is usually lower than with *P. pastoris*. 

Baculovirus-infected insect cells have been used as an alternative system to *Xenopus * oocytes for expression and characterization of plant channel proteins. Several channel proteins which were not functional in oocytes could be characterized in baculovirus-infected insect cells such as the K^+^ channel proteins AKT1 [93], KAT1 [94], KCO1 [95] from Arabidopsis, and KST1 [96] and SKT1 [97] from potato. 

To investigate the interaction between AUX1 and its transport substrate indole-3-acetic acid (IAA) from Arabidopsis, an epitope-tagged version of AUX1 was expressed at high levels in a baculovirus expression system and suitable membrane fragments were prepared from baculovirus-infected insect cells for direct measurement of IAA binding to AUX1. AUX1-IAA interactions were determined using a radio-ligand binding assay to confirm that AUX1 was able to bind IAA with an affinity (Kd) of 2.6 mM, comparable with estimates of the Km for IAA transport [98]. 

The main disadvantages of using baculovirus-infected insect cells are difficulties in constructing the expression vectors, requirements for more complex laboratory facilities and skills, and the short expression periods after infection. 

#### 3.2.3. Xenopus Laevis Oocytes

The oocytes of the South African clawed frog, *Xenopus laevis*, are also used for heterelogous expression of eukaryotic genes. The mRNA for the target protein, introduced by microinjection into the cytoplasm, is translated and the protein is posttranslationally modified by the oocyte [99]. Direct injections of DNA into the nucleus are also possible, but the manipulations are difficult as the nucleus can easily be damaged in the process.

Investigations on membrane transport proteins can be readily performed on oocytes where techniques for electrophysiological measurements are well established. Although, a high proportion of cells express the foreign gene after injection variations in the quality of oocytes and in the ability of individual cells to produce the heterelogous protein can cause problems. Oocytes are not suitable for preparing large quantities of proteins and the short expression period often leads to technical difficulties. The system can also not be sustained over long periods of time and is not suitable for stable expression [99, 100].

*Xenopus* oocytes have, however, provided a powerful heterologous expression system for animal as well as plant genes. The possibility of using *Xenopus * oocytes as heterologous expression systems for the identification of plant transporters was first demonstrated by the expression of the H^+^/glucose transporter STP1 from Arabidopsis [101]. It has, since, been mainly used for production of transporters including potassium channels, H^+^/hexose cotransporters, aquaporins, and chloride channels [99]. In addition, functional expression of a nitrate transporter [102], a K^+^/Na^+^ transporter [39, 103, 104], ammonium transporters [105, 106], sucrose transporters [107–109], Al-activated malate transporters [110, 111], polyol transporters [112, 113], inositol transporters [114, 115], an amino acid transporter [116], a cation–Cl-cotransporter [117], and an anion-selective transporter [118] in *Xenopus* oocytes were investigated. Cases where channel proteins expressed in oocytes were not functional have also been reported. These include the K^+^ channels AKT1 from Arabidopsis [93, 119, 120], TaAKT1 from wheat [121], DKT1 from carrot [122], and OsAKT1 from rice [123]. The causes for the lack of function of these recombinant proteins are not clear. 

Several studies have used expression of a wild type and its mutant forms in *Xenopus* oocytes to confirm the in vivo functions of plant proteins, especially transporters and plasma membrane intrinsic proteins (PIPs or aquaporins). To demonstrate whether or not the plant K^+^ channels form multimers, the wild type and a mutant were coexpressed in *Xenopus * oocytes [120]. Coexpression of tomato ammonium transporter (LeAMT1;1) and its mutant in *Xenopus* oocytes inhibited ammonium transport, suggesting homooligomerization [105]. In another study, the role of phosphorylation in the water channel activity of wild-type and mutant ZmPIP2;1 was studied in *Xenopus* oocytes [124].

In recent studies, the *Xenopus* oocyte expression system was used to investigate structure-function relationships. In one example, differences in the function of two cation transporters, wheat HKT1 and Arabidopsis AtHKT1, were investigated using a series of AtHKT1/HKT1 chimeras with point mutations [103]. 

## 4. Conclusions

Heterologous expression of plant genes in other host organisms has two main applications: (1) overexpression of the encoded protein, for biochemical and biophysical characterization and (2) expression of foreign genes for determination of the function of the encoded protein by complementing in a mutant host. Overexpression of recombinant proteins is usually carried out with a cleavable tag to simplify purification in large quantities. In contrast, complementation studies are carried out in null mutants to restore a missing activity in vivo.

Decisions on which expression vectors to use and the choice of the expression host depend on the particular application. In general *E.coli* is the first choice as host because of its simplicity, availability of expression vectors, cost effectiveness, and availability of extensive genetic information on this host. Alternative expression systems are used only if the recombinant protein is inactive due to lack of essential posttranslational modifications and when detailed studies on the recombinant protein function are planned. Yeast systems have the advantage of ease of manipulation and short generation time. *S. cerevisiae* has been extensively used for functional complementation, biochemical, and electrophysiolagical characterization of plant membrane and transporter proteins. *P. pastoris* is the preferred host for overexpression of several plant enzymes. Baculovirus-mediated insect cell expression offers the possibility for detailed investigations of plant enzymes and transporters. The oocyte from *Xenopus laevis* is often used for monitoring activity and biochemical and electrophysiological characterization of plant plasma membrane transporter and pump proteins. 

 Heterologous expression is a powerful tool for functional and biochemical analyses of genes and gene families isolated from various organisms. It is particularly important for plants where the whole genome sequence is not available. This system will also provide *denovo* analysis. Its limitations, however, should be kept in mind, especially when interpreting the results in terms of the native structure and function of proteins. Major problems arise from misfolding and mislocalization of recombinant proteins in foreign hosts. Strategies developed to avoid misfolding of recombinant proteins include expression in periplasmic space, expression with a tag, and utilization of different hosts. Mislocalization, on the other hand, may occur because the recombinant protein may take over the function of the missing host protein [170]. Conclusions on function need to be tested in alternative hosts and eventually in the plant itself.

## Figures and Tables

**Table 1:** Heterologous expression of plant proteins grouped according to the host cells.

Protein expressed	Plant	Reference
**Escherichia coli**		
Lipase B (PalB)	*Pseudozyma antarctica*	[18]
Oxalate oxidase	*Hordeum vulgare, Triticum aestivum*	[21]
Osmotin-like cryoprotective protein	*Solanum dulcamara*	[22]
Thaumatin-like protein (ATLP3)	*Arabidopsis thaliana*	[23]
Osmotin-like protein (SnOLP)	*Solanum nigrum *	[24]
RHG1-LRR domain	*Glycine max*	[25]
Chloroplast transglutaminase (TGZ)	*Zea mays*	[26]
FatB thioesterase	*Madhuca butyracea *	[27]
Glutamatecysteine ligase (GCL)	*Arabidopsis thaliana*	[28]
DELLA proteins	*Arabidopsis thaliana, Malus domestica *	[29]
K^+^ transporters; KAT1, AKT2-3, AtKUP1/AtKT1/AtPOT1, AtKUP2/AtKT2/AtPOT2, AtHKT1	*Arabidopsis thaliana*	[30–32]
K^+^ transporters, EcHKT1, EcHKT2	*Eucalyptus globulus *	[33]
ATP/ADP transporter	*Arabidopsis thaliana*	[34]
HAK K^+^ transporters, CnHAK1,CnHAK2	*Cymodocea nodosa*	[35]
Peptide transporter family member, AgDCAT1	*Alnus glutinosa*	[36]
Type 1 MT, dMT	*Triticum durum*	[37]
Type 1 and Type 2 MTs	*Vicia faba*	[38]
MT1, MT2, and MT3	*Arabidopsis thaliana*	[39]
Type 3 MT3-A	*Elaeis guineensis *	[40]
Type 2 MT, QsMT	*Quercus suber*	[41]
Soybean seed ferritin	*Glycine max*	[125]
***Saccharomyces cerevisiae ***		
H^+^-amino acid symporter and K^+^ channel, KATl	*Arabidopsis thaliana*	[47]
Phosphate transporters; AtPT1 and AtPT2	*Arabidopsis thaliana*	[48]
K^+^transporter, HvHAKI	*Hordeum vulgare*	[49]
K^+^ transporters, AtKT1, and AtKT2, AtKUP1	*Arabidopsis thaliana*	[50, 51]
K^+^ transporter, HKT1	*Triticum aestivum*	[52, 53]
Sulfate transporters, LeST1-1 and LeST1-2	*Lycopersicon esculentum*	[54]
Copper transporters, (COPT1–5)	*Arabidopsis thaliana*	[55]
Peptide transporter, AtPTR1	*Arabidopsis thaliana*	[56]
K^+^/H^+^ antiporter, *At*Chx17	*Arabidopsis thaliana*	[57]
Hexose transporters, VvHT4 and VvHT5	*Vitis vinifera*	[58]
Plasma membrane-localized H^+^/inositol symporter, AtINT2	*Arabidopsis thaliana*	[59]
High affinity GABAtransporter, AtGAT1	*Arabidopsis thaliana*	[60]
Tonoplast Intrinsic Proteins, AtTIP2;1 and AtTIP2;3	*Arabidopsis thaliana*	[61]
Sorbitol transporters, PmPLT1 and PmPLT2	*Plantago major*	[62]
***Pichia pastoris ***		
Nitrate reductase	*Spinacia oleracea,* *Zea mays*	[69]
Invertase	*Ipomoea batatas*	[70]
*α*1,6-galactosyltransferase	*Trigonella foenum-graecum*	[71]
*α*1,6-xylosyltransferase	*Arabidopsis thaliana*	[72]
Glycosyltransferases	*Arabidopsis thaliana Bos taurus, Drosophila melanogaster, Caenorhabditis elegans, Leucopersicon esculentum*	[73]
*β*-D-fructofuranosidase	*Oryza sativa*	[74]
Apyrase	*Solanum tuberosum*	[75]
Oxalate oxidases, HvOXO, TaOXO	*Hordeum vulgare, Triticum aestivum*	[76, 77]
Lectin	*Canavalia brasiliensis, Nicotiana tabacum*	[2, 79]
Low-affinity cation transporter (LCT1)	*Triticum aestivum*	[80]
2S albumin storage proteins (AL1 and AL3)	*Glycine max*	[126]
**Baculovirus-mediated insect cell**		
Patatin	*Solanum tuberosum*	[81]
Reductase isoforms, AR1 and AR2	*Arabidopsis thaliana*	[82]
Peroxisomal short-chain acyl-CoA oxidase A	*Arabidopsis thaliana*	[83]
Cyclin-dependent kinase A (CDKA)	*Arabidopsis thaliana*	[84]
NADH-cytochrome (Cyt) b5 reductase	*Arabidopsis thaliana*	[85]
Geranylgeranyltransferase-I (GGT-I)	*Arabidopsis thaliana*	[86]
Acyl-CoA synthetase	*Arabidopsis thaliana*	[87]
Homogentisate phytyltransferase	*Arabidopsis thaliana *	[88]
(+)-Abscisic Acid 8’-Hydroxylase	*Arabidopsis thaliana *	[89]
*β*1,2-xylosyltransferase	*Arabidopsis thaliana*	[90]
Ethylene-inducing xylanase	*Nicotiana tabacum*	[91]
ADP-glucose pyrophoshorylase (AGPase)	*Hordeum vulgare*	[92]
K^+^ channels, AKT1, KAT1, and KCO1	*Arabidopsis thaliana *	[93–95]
K^+^ channels KST1, SKT1, and KST1	*Solanum tuberosum*	[96, 97]
Transporter AUX1	* Arabidopsis thaliana *	[98]
*β*-phaseolin polypeptides	*Phaseolus vulgaris*	[127]
Ac-specific ORFa protein,	*Zea mays*	[128]
Cysteine protease papain	*Carica papaya*	[129]
Mitochondrial protein URF13	*Zea mays*	[130]
LAT52 protein	*Lycopersicon esculentum*	[131]
Auxin-binding protein (ABP1)	*Zea mays, Nicotiana tabacum*	[132, 133]
Calreticulin and auxin binding protein	*Zea mays*	[134]
Cinnamate 4-Hydroxylase	*Arabidopsis thaliana*	[135]
Cryptochrome-1	*Arabidopsis thaliana*	[136]
Phototropin 2	*Arabidopsis thaliana*	[137]
Histidinol dehydrogenase	*Brassica oleracea*	[138]
Putative soluble epoxide hydrolase (sEH)	*Solanum tuberosum*	[139]
lmidazoleglycerolphosphate dehydratase	*Arabidopsis thaliana*	[140]
Phytone synthase, Phytoene desaturase	*Narcissus pseudonarcissus*	[141, 142]
4-coumarate:coenzyme A ligase (4Cl)	*Populus trichocarpa, Populusdeltoides *	[143]
**Xenopus** **laevis** **oocytes**		
Na^+^ − K^+^cotransporter HKT1	*Arabidopsis thaliana*	[39]
AgDCAT1 nodule-specific transporter	*Alnus glutinosa*	[43]
AtNAR2.1/AtNRT2 Nitrate Transport System	*Arabidopsis thaliana*	[102]
HKT Constructs, AtHKT1_HKT1 chimeras	*Triticum aestivum*, *Arabidopsis thaliana *	[103]
HKT1 superfamily of K^+^/Na^+^ transporters	*Eucalyptus camaldulensis *	[104]
Ammonium transporter, LeAMT1	*Lycopersicon esculentum*	[105]
Ammonium transporter, AtAMT1;2	*Arabidopsis thaliana*	[106]
Sucrose transporters, AtSUC2, AtSUC9, LjSUT4	*Arabidopsis thaliana,* *Lotus japonicus*	[107–109]
Al-activated malate transporter, BnALMT1,BnALMT2, ALMT1	*Brassica napus, Triticum aestivum*	[110, 111]
Polyol transporters, AtPLT5, PmPLT1	*Arabidopsis thaliana, Plantago major*	[112, 113]
Inositol transporter2, AtINT2, AtINT4	*Arabidopsis thaliana*	[114, 115]
Amino acid transporter, AtCAT6,	*Arabidopsis thaliana*	[116]
Cation–Cl- cotransporter, CCC	*Arabidopsis thaliana*	[117]
Anion-selective transporter, ZmALMT1	*Zea mays*	[118]
K^+^channel, SIRK	*Vitis vinifera*	[144]
K^+^ channel, KZM1	*Zea mays*	[145]
K^+^ channel, ZMK1	*Zea mays*	[146]
K^+^ channels, SKT1 and LKT1	*Solanum tuberesum*, *Lycopersicon esculentum *	[147]
AKT2-KAT2 subunitits	*Arabidopsis thaliana*	[148]
*K* ^+^ channel, KAT1	*Arabidopsis thaliana*	[149]
Cyclic nucleotide-gated ion channels AtCNGC2, AtCNGC1, -2	*Arabidopsis thaliana*, *Nicotiana tobacum *	[150, 151]
Putative transporter (GmN70)	*Glycine max*	[152]
Al-activated malate transporter, TaALMT1	*Triticum aestivum*	[153]
High affinity *γ*-aminobutyric acid transporter, AtGAT1	*Arabidopsis thaliana*	[154]
Aquaporins, ZmPIP1a, ZmPIP1b, ZmPIP2a, PIP1, ZmPIP2;1	*Zea mays*	[124, 155, 156]
Aquaporin, PIP1	*Lycopersicon esculentum*	[157]
Aquaporin, PIP2	*Juglans regia*	[158]
Tonoplast intrinsic protein, AtTIP2;1	*Arabidopsis thaliana*	[159, 160]
Aquaporin, McTIP1;2	*Mesembryanthemum crystallinum*	[161]
Aquaporin, HvPIP1;6	*Hordeum vulgare*	[162]
Tonoplast intrinsic protein, PgTIP1	*Panax ginseng*	[163]
Nodulin 26 intrinsic protein, AtNIP2;1	*Arabidopsis thaliana*	[164]
PIP-1-type; NtPIP1;1, NtAQP1; PIP-2-type; NtPIP2;1	*Nicotiana tabacum*	[165]
CjMDR1, ATP-binding cassette protein	*Coptis japonica*	[166]
GlpF-like intrinsic protein (GIP1;1),	*Physcomitrella patens*	[167]
Metal tolerance protein1, AtMTP1	*Arabidopsis thaliana*	[159]
AtTPK4 tandem-pore K^+^channel	*Arabidopsis thaliana*	[168]
FRD3, multidrug and toxin efflux (MATE)	*Arabidopsis thaliana*	[169]
